# Clinicopathological features and outcomes in gastric-type of HPV-independent endocervical adenocarcinomas

**DOI:** 10.1186/s12885-021-08792-7

**Published:** 2021-10-11

**Authors:** Lili Chen, Yizhen Niu, Xiaoyun Wan, Lina Yu, Xiaofei Zhang, Amanda Louise Strickland, Liya Dong, Feng Zhou, Weiguo Lu

**Affiliations:** 1 Department of Gynecologic Oncology, Women’s Hospital, School of Medicine, Zhejiang University, Hangzhou, Zhejiang China; 2 Women’s Reproductive Health Research Laboratory of Zhejiang Province, Women’s Hospital, School of Medicine, Cancer Center, Zhejiang University, Hangzhou, Zhejiang China; 3 Department of Pathology, Women’s Hospital, School of Medicine, Zhejiang University, Province, Zhejiang, Hangzhou China; 4 Department of Pathology, Northwestern University, Feinberg School of Medicine, Chicago, IL USA

**Keywords:** HPV-independent endocervical adenocarcinoma (HPVI ECA), Gastric-type, Clinicopathological features, Serum CA19–9

## Abstract

**Background:**

We aimed to analyze the clinicopathological features and outcomes of patients with gastric-type of HPV-independent endocervical adenocarcinoma (GAS HPVI ECA), and compare them with non-GAS HPVI ECA cases.

**Methods:**

Thirty-eight GASs [including 17 minimal deviation adenocarcinoma (MDA), 21 non-MDA GAS] and 17 non-GAS HPVI ECAs were studied. Data of clinical features, pathological characteristics, treatment, and outcomes were evaluated.

**Results:**

The median age of patients with GAS and non-GAS HPVI ECA was 46 and 48 years, respectively (*p* = 0.93). Compared with non-GAS HPVI ECAs, GAS had more common complains of vaginal watery discharge (*p* = 0.04). GAS cases were also associated with higher clinical stage (*p* = 0.036), more common in deeper cervical stromal invasion (*p* = 0.002) and lymphoavascular invasion (*p* = 0.044). GAS was associated with worse median progression-free survival (PFS) (*p* = 0.02) and median overall survival (OS) (*p* = 0.03) over patients with non-GAS HPVI ECAs. MDA had similar clinical and pathological features and prognosis compared with non-MDA GAS. Of note, serum CA19–9 levels were significantly higher in GAS than that in non-GAS HPVI ECA cases.

**Conclusions:**

GAS cases were more likely to have high risk pathological factors and poorer PFS and OS compared with non-GAS HPVI ECAs. Serum CA19–9 may be helpful for diagnosis and screening in patients with GAS.

**Supplementary Information:**

The online version contains supplementary material available at 10.1186/s12885-021-08792-7.

## Background

Endocervical adenocarcinomas (ECAs) comprise up to 25% of all cervical cancers [1–3], and are frequently related to persistent infection of human papillomavirus (HPV) 16/18/45 [4]. There is also a small subtype of non-HPV-associated ECAs [1, 3, 5, 6]. Unlike HPV-associated ECAs, non-HPV-associated ECAs are frequently located in the upper endocervix, resulting in missed detection or misdiagnosis [7, 8]. According to the 2020 World Health Organization (WHO) Classification of Female Genital Tumors [9], ECAs are subclassified into HPV-associated (HPVA) and HPV-independent (HPVI) groups. HPVI ECAs include gastric type ECA (GAS) [including minimal deviation adenocarcinoma (MDA)], endometrioid carcinoma (EMCA), clear cell adenocarcinoma (CCC), mesonephric carcinoma (MC), and adenocarcinoma, not otherwise specified (NOS). First described by Japanese groups [10–12], GAS, including MDA, is the second most common subtype of ECA and the most common subtype of HPVI ECAs [13]. Although considered rare in Western countries [14, 15], GAS accounts for up to 25% of all ECAs in Asian population [11, 16]. Other types of HPVI ECAs are rare, and data about their clinical behavior is limited.

GASs are frequently present with an advanced stage, poor prognosis, and diverse clinical features with different subtypes [11, 15]. However, these cases are rare and large clinicopathologic studies in this field are limited. Here, we conduct a relatively large retrospective analysis focused on the clinicopathological features and outcome of GASs to provide a useful reference for the diagnosis and treatment of such tumors.

## Methods

### Case selection

Patients with a final diagnosis of GAS and non-GAS HPVI ECA from 2014 to 2020 in our hospital were identified. Hematoxylin and eosin (H&E) and immunohistochemical (IHC) staining slides were reviewed by 2 gynecologic pathologists (F.Z. and X.Z.) in a blinded fashion and the pathologic diagnosis was confirmed. All related clinical data including age, symptoms, imaging materials, level of serum CA19–9, treatment, clinical outcome were collected from the electronic clinical information system database. All tumors were classified according to the 2020 WHO Classification of Female Genital Tumors. Patients were clinically staged using the 2018 International Federation of Gynecology and Obstetrics (FIGO) system. The results of Thinprep cytologic test (TCT), high-risk HPV (hrHPV) (tested by Aptima, Cervista or Hybrid capture 2 assay), and p16 performed as part of clinical care were recorded. Informed consent was obtained from all subjects involved in the study. IRB approval was obtained by the Ethics Committee of our hospital.

### Serum CA19–9 examination

Serum CA19–9 was estimated by using an automated chemiluminescence analyzer (Shanghai Roche Diagnostic products Co., LTD., China). All assay procedures were performed based on manufacturer instructions. The normal upper limitation is 39 U/ml.

### Statistical analysis

Non-normal distributed parametric variables and categorical data were separately compared by the Mann–Whitney U-test and Chi-square test in the IBM SPSS software environment (version 26.0). The Kaplan-Meier method was used to generate survival curves and the log-rank method was used for statistical testing. Survival curves and the log-rank test were both performed using the R software (version 4.1.0; www.r-project.org). Progression-free survival (PFS) was calculated from the date of diagnosis to the date of tumor recurrence, progression, or death. The overall survival (OS) was defined as the time between the date of surgery and the last date of follow-up or death from any cause. Two-sided *p* values were reported. p values less than 0.05 were considered statistically significant.

## Results

### Clinicopathological features of patients

**Fig. 1:**
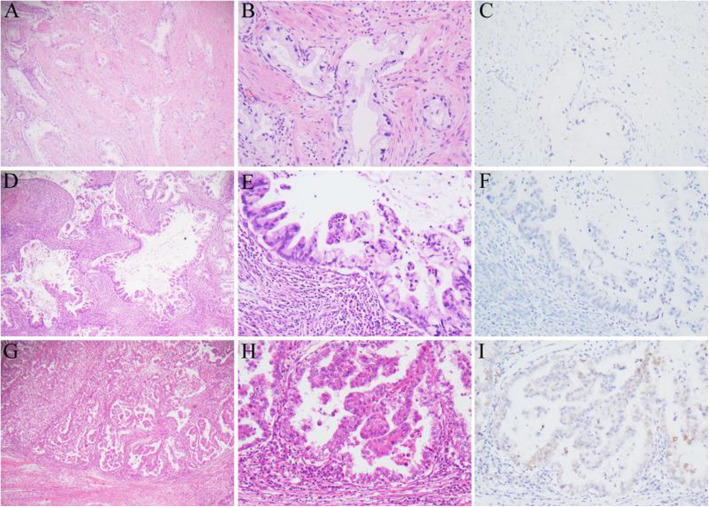
Examples of different histological types of HPV independent endocervical adenocarcinoma. (A-C) Minimal deviation adenocarcinoma (MDA): (A) Well-formed glands diffusely infiltrating cervical wall (H&E, 50 x). Neoplastic cells with clear and voluminous cytoplasm and basally located nuclei with mild cytologic atypia (H&E, 200 x). (C) Negative p16 expression in tumor cells. (D-F): Moderately differentiated gastric type adenocarcinoma: (D) Papillary proliferation of non-MDA gastric type adenocarcinoma (H&E, 50 x). (E) Neoplastic cells with moderate cytologic atypia and abundant clear cytoplasm showing distinct cell borders (H&E, 200 x). (F) Negative p16 expression in tumor cells. (G-I) Clear cell carcinoma: (G) Neoplastic glands infiltrating fibromatous stroma (H&E, 50 x). (H) Tumor cells with clear cytoplasm and severe nuclear atypia (H&E, 200 x). (I) Patch p16 expression in tumor cells

From 2014 to 2020, totally 512 cases were diagnosed with ECA or ECA in situ. Fifty-five cases (10.7%) were confirmed as HPVI ECA. The specimens of HPVI ECA included 47 hysterectomies, 2 cone excisions, and 6 biopsies. Among them, 38 cases were GAS (including 17 MDA, 21 non-MDA GAS) and 17 were non-GAS HPVI ECAs (including 12 CCC, 4 EMCA, and 1 MC) (Fig. 1). 55 of 55 (100%) HPVI ECAs were p16 negative or patchy positive. hrHPV test results were available for 41 of 55 (74.5%) HPVI ECAs, all of which were negative. The remaining 14 HPVI ECAs (7 GAS and 7 CCC) had no records of hrHPV testing but with negative or patchy positive p16 results. Patient pathologic diagnosis, p16 status, and HPV status information are summarized in Table S1.

### Clinical features of GAS and compared to non-GAS HPVI ECA

The median age of patients with GAS was 46 years old (IQR: 41.8, 59.3), with no significant difference compared with that of patients with non-GAS HPVI ECA (48 years, IQR: 40.5, 59.0, *p* = 0.93). 27 patients with GAS had clinical symptoms, with 18/27 (66.7%) complained of irregular bleeding or contact bleeding and 9/27 (33.3%) experienced vaginal watery discharge. Meanwhile, 14 patients with non-GAS HPVI ECAs had clinical symptoms, in which 100% patients had irregular vaginal bleeding or contact bleeding. Vaginal watery discharge was more frequently noted in GAS cases than in non-GAS HPVI ECAs (*p* = 0.04). Combined pelvic examination with imaging data, although there was no significant difference in lesion size of the greatest dimension between the two groups, 17 patients (44.7%) in the GAS group had a tumor size larger than 4 cm, while only 4 patients (23.5%) in the non-GAS HPVI ECA group had a tumor size larger than 4 cm (*p* = 0.24). This information is illustrated inTable1.

Twenty-six of GAS cases had TCT results, with 10/26 (38.5%) were negative for intraepithelial lesions or malignancy (NILM). There was no difference in positivity rate of TCT between GAS (61.5%) and non-GAS HPVI ECA type (5/9, 55.6%, *p* = 0.87). Seen Table 1.

**Table 1:** Comparing of clinical features between cervical gastric-type and non-gastric type adenocarcinoma

Clinical features	GAS	Non-GAS	P value
Age (median, IQR)	46 (41.8,59.3)	48 (40.5,59.0)	0.93
Symptoms (n, %)			0.04
Bleeding	18 (66.7%)	14 (100%)	
Watery discharge	9 (33.3%)	0 (0%)	
Tumor Size in the largest dimension (n, %)			0.24
< 4 cm	21 (55.3%)	13 (76.5%)	
≥ 4 cm	17 (44.7%)	4 (23.5%)	
TCT (n, %)			0.87
NILM	10 (38.5%)	4 (44.4%)	
Abnormal	16 (61.5%)	5 (55.6%)	
Stage (n, %)			0.036
I-IIA	17 (46.0%)	11 (84.6%)	
IIB-IV	20 (54.0%)	2 (15.4%)	
Lymph nodes metastasis (n, %)			0.23
Negative	24 (70.6%)	12 (92.3%)	
Positive	10 (29.4%)	1 (7.7%)	
Deep stromal invasion (n, %)			0.002
Negative	4 (11.8%)	8 (61.5%)	
Positive	30 (88.2%)	5 (38.5%)	
LVSI (n, %)			0.044
Negative	19 (55.9%)	12 (92.3%)	
Positive	15 (44.1%)	1 (7.7%)	

### GAS was associated with worse pathological risk factors, higher stage, and poorer prognosis compared to non-GAS HPVI ECA

In total, 47 hysterectomies (34 GAS and 13 non-GAS HPVI ECAs) were available for analyzing ancillary factors. The incidence of deep stromal invasion and lymphovascular space invasion (LVSI) in GAS were significantly higher than that of non-GAS HPVI ECAs (88.2% vs 38.5%, *p* = 0.002; 44.1% vs 7.7%, *p* = 0.044, respectively). The incidence of lymph nodes metastasis in GAS was also higher than that in non-GAS HPVI ECAs, although no statistical difference was found (29.4% vs 7.7%, *p* = 0.23). Seen Table 1.

Among the 50 HPVI ECAs (37 GAS and 13 non-GAS HPVI ECAs) with clinical pathological staging information, 17/37 (46.0%) were staged as I-IIA and 20/37 (54.0%) were staged as IIB-IV for GAS. Meanwhile, for non-GAS HPVI ECAs, 11/13 (84.6%) were staged I-IIA and 2/13 (15.4%) was staged IIB-IV. Here we used IIA as the boundary to state which type of HPVI ECA is more likely to infiltrate into the parametrial, pelvic cavity or have distant metastasis. Patients with GAS were more likely to have an advanced clinical stage by infiltration into parametrial and remote organs compared with those of non-GAS HPVI ECAs (*p* = 0.036).

In GAS, 36 of 38 patients underwent surgery. Except for one patient who underwent a radical trachelectomy, the remaining 35 were all treated with radical hysterectomy and lymphadenectomy. 31/36 (81.6%) received radiotherapy (RT)/chemotherapy (CT) as post-operative adjuvant treatment. Meanwhile, 5/38 (13.2%) received surgery only. 2/38 (5.3%) received concurrent chemo-radiotherapy without surgery. In non-GAS HPVI ECAs, 15 of 17 patients received radical hysterectomy with pelvic lymphadenectomy and 2/17 (11.8%) received RT and CT without operation. Of the patients with surgical management, 13/15 (86.7%) received adjuvant RT/CT (Table S2).

**Fig. 2:**
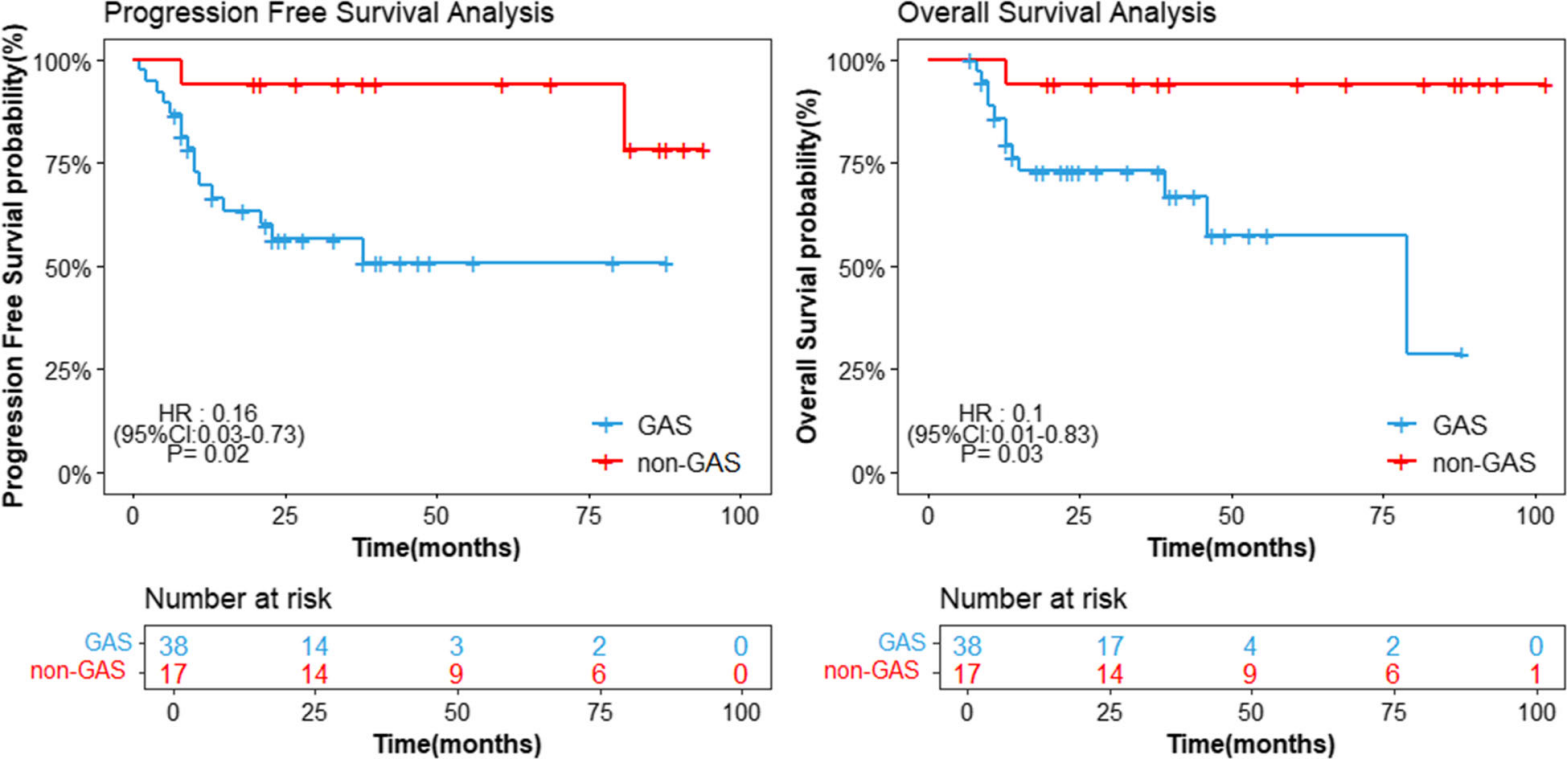
Kaplan-Meier progression free survival (PFS) and overall survival (OS) analysis for GAS vs. non-GAS. Abbreviations: GAS = gastric type ECA

Complete follow-up information was available and included in a survival analysis for 55 patients with HPVI ECA. In the GAS group, 16 patients relapsed, with lesions in the pelvic cavity, vaginal stump, great omentum metastasis, intestinal metastasis, pulmonary metastasis, etc. A total of 12 patients died. In the non-GAS HPVI ECA group, 2 cases recurred and 1 case died as of the last follow-up. GAS patients showed significant reduction in the median PFS over patients in the non-GAS HPVI ECA group with hazard ratio (HR) 0.16 (95% CI: 0.03, 0.73), *p* = 0.02. And GAS patients also showed significant reduction in the median OS over patients in the non-GAS HPVI ECA group with HR 0.10 (95% CI: 0.01, 0.83), *p* = 0.03(Fig. 2).

### Comparison of clinicopathological features and outcomes between MDA and non-MDA GAS

**Fig. 3:**
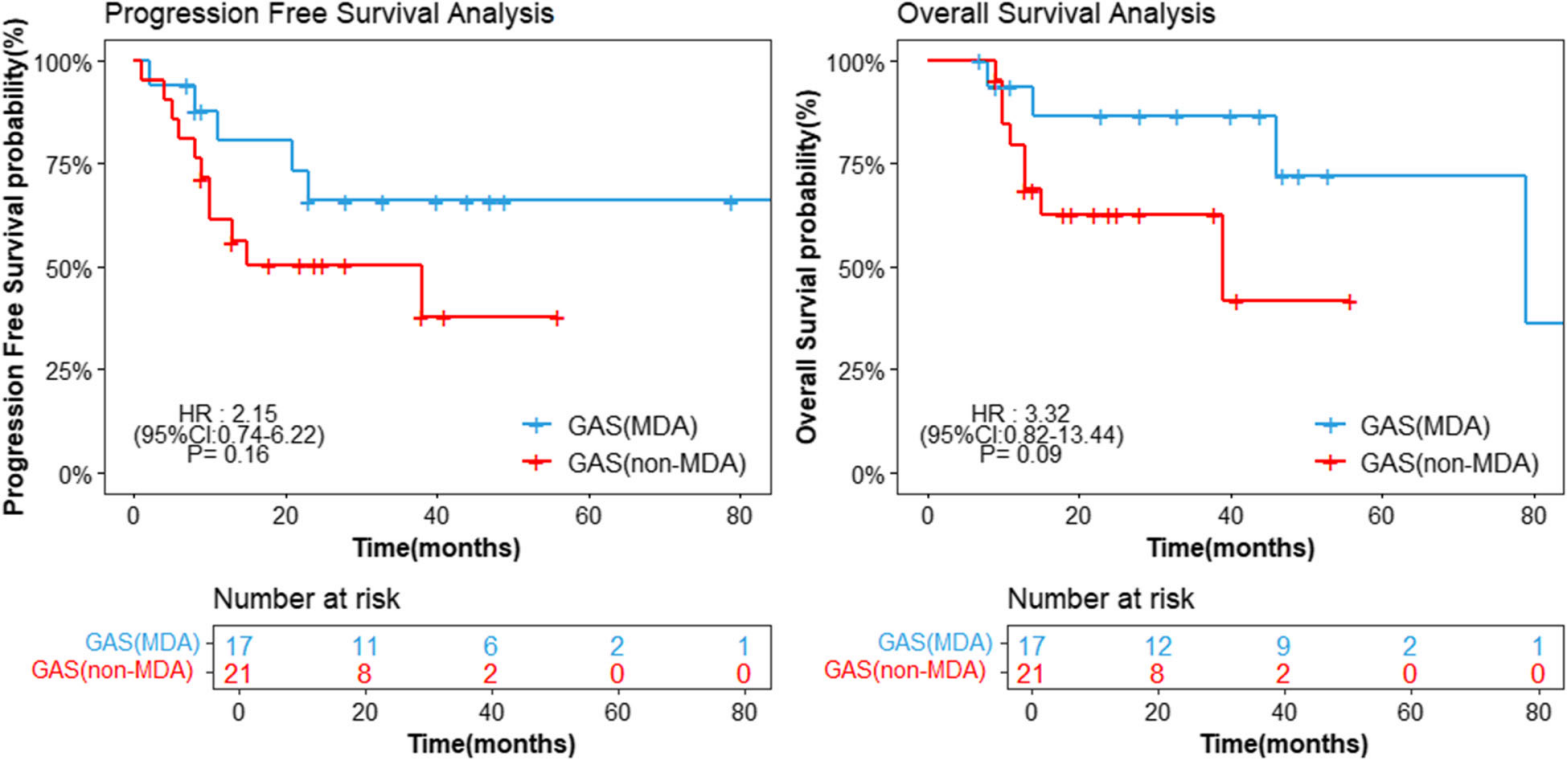
Kaplan-Meier progression free survival and overall survival estimates for MDA vs. non-MDA. Abbreviations: MDA = minimal deviation ECA

In GAS cases, 17/38 (44.7%) were diagnosed as MDA and 21/38 (55.3%) were diagnosed as non-MDA GAS. There were no statistical differences between the two groups in age (*p* = 0.86), symptoms (*p* = 0.34), and tumor sizes (*p* = 0.56). Although the cytological results of TCT with NILM were more common in MDA group than that in non-MDA GAS group, no significantly difference was found (63.0% vs 20.0%, *p* = 0.06). Additional histologic risk factors were also evaluated, with lymph node metastasis (*p* = 0.95), deep stromal invasion (*p* = 0.43), and LVSI (*p* = 0.44) were all similar between MDA and non-MDA GAS (Table 2). A total of 5 cases relapsed and 4 case died during the follow-up in MDA patients. In non-MDA GAS patients, 11 cases reoccurred, and 8 cases died. There were no significant differences of median PFS [HR: 2.12, 95% CI (0.74, 6.22), *p* = 0.16] and median OS [HR:3.32 (95% CI: 0.82, 13.44), *p* = 0.09] between MDA and non-MDA GAS groups (Fig. 3).

**Table 2:** Comparison of Clinical features between MDA and non-MDA GAS

Clinical features	MDA	Non-MDA	P value
Age (median, IQR)	46 (42.0, 62.0)	48 (40.5, 59.0)	0.86
Symptoms (n, %)			0.34
Bleeding	7 (53.8%)	11 (78.6%)	
discharge	6 (46.2%)	3 (21.4%)	
Tumor Size in the greatest dimension (n, %)			0.56
< 4 cm	8 (47.1%)	13 (61.9%)	
≥ 4 cm	9 (52.9%)	8 (38.1%)	
TCT (n, %)			0.06
NILM	7 (63.6%)	3 (20.0%)	
Abnormal	4 (36.4%)	12 (80.0%)	
Stage (n, %)			0.83
I-IIA	8 (47.1%)	9 (45.0%)	
IIB-IV	9 (52.9%)	11 (55.0%)	
Lymph node metastasis (n, %)			0.95
Negative	10 (66.7%)	14 (73.7%)	
Positive	5 (33.3%)	5 (26.3%)	
Deep stromal invasive (n, %)			0.43
Negative	3 25.0%)	1 (5.3%)	
positive	12 (75.0%)	18 (94.7%)	
LVSI (n, %)			0.44
Negative	10 (66.7%)	9 (47.4%)	
positive	5 (33.3%)	10 (52.6%)

### GAS was associated with higher level of serum CA19–9 compared to non-GAS HPVI ECA

We compared the level of serum CA19–9 among MDA, non-MDA GAS, and non-GAS HPVI ECA types. The median level of CA199 was 161.0 U/ml (IQR: 17.2, 712.14) in MDA type, 22.8 U/ml (IQR: 12.5, 176.0) in non-MDA GAS type, and 10.1 U/ml (IQR: 6.3, 40.4) in non-GAS HPVI ECA type. The level of CA199 in MDA type was not significantly higher compared with the non-MDA GAS group (*p* = 0.121). Compared with the non-GAS HPVI ECA group, the CA199 levels in MDA and non-MDA GAS types were all significantly higher (*p* = 0.001 and *p* = 0.024). The number of patients with abnormal CA19–9 levels (> 39 U/ml) in different groups was also analyzed. The cases with elevated CA19–9 were more in MDA group than that in the non-MDA GAS group (*p* = 0.022) and non-GAS HPVI ECA group (*p* = 0.006), while no significant difference was found between non-MDA GAS and non-GAS HPVI ECA types. Seen Table 3.

**Table 3:** Comparing of levels and cases of serum CA199 among MDA, non-MDA and non-GAS

CA199	MDA	non-MDA	non-GAS
Median(U/ml), (IQR)	161.0 (17.15,712.14)	22.8 (12.5,176.0)	10.1 (6.4,40.4)
p		0.121^†^	0.001^‡^ 0.024^※^
	n, %	n, %	n, %
< 39 U/ml	5 (29.4%)	14 (66.7%)	13 (76.5%)
≥39 U/ml	12 (70.6%)	7 (33.3%)	4 (23.5%)
p		0.022^†^	0.006^‡^ 0.45^※^

^†^Median levels and cases of MDA compared with non-MDA, ^‡^Median levels and cases of MDA compared with non-GAS. ^※^Median levels and cases of non-MDA compared with non-GAS

## Discussion

This is a relatively large retrospective study of GASs, which were confirmed by clinicopathological characteristics including p16 and hrHPV results. In our study, GAS cases had a different constellation of clinical presentation and laboratory results compared with non-GAS HPVI ECA cases, including vaginal watery discharge and elevated serum CA19–9. In addition, GAS cases were more likely to have deep cervical stromal invasion, LVSI and an advanced stage when compared against those of non-GAS HPVI ECAs. Finally, GAS cases were more commonly found to have prognosis with poorer PFS and OS.

In this series of HPVI ECA cases, GAS accounted for 69.1% of all studied cases, followed by CCC (21.8%). The prevalence of different histologic types was similar to Stolnicu et al.’s report [13], in which they studied 40 cases of HPVI ECAs. Of those 40 cases, GAS and CCC accounted for 67.3 and 20%, respectively. Stolnicu et al. [13] reported patients with non-GAS HPVI ECA tended to be older. However, the ages of different subtypes of HPVI ECA were similar in other reports [17, 18]. These discrepant results may be related to the limited number of cases of these studies. In our cohort, the ages of patients with GAS or non-GAS HPVI ECAs were similar, with the median age being 46 (IQR: 43.5, 62) in MDA, 43 (IQR: 38.5, 58) in non-MDA GAS, and 48 (IQR: 40.5, 59) in non-GAS HPVI ECAs.

Unlike usual type HPVA ECAs, GAS is frequently located in the upper endocervix and present with a bulky cervix [7, 8]. Because the number of such cases is relatively limited, reports about the comparison of clinical characteristics, pathological features and outcomes between GAS and non-GAS HPVI ECA are rare [11, 15]. According to our findings, the clinical manifestations of GAS included vaginal watery discharge and/or bleeding, while patients with non-GAS HPVI ECA mostly complained of vaginal bleeding. Consensus guidelines for management of cervical dysplasia in the screening setting have not yet been reached to accommodate the three most widely available screening strategies: primary HPV testing, co-testing with HPV testing and cervical cytology, and cervical cytology alone [19]. This is a critical need for these guidelines because HPVI ECAs are negative for hrHPV, and cytology results become more important especially in those without abnormal appearance of cervix. According to previous reports, the positivity rate of TCT screening in ECAs is 40–50%, which is much lower than that in SCCs (above 90%) [20]. Nakamura et al. [21] reported 78% NILM of TCT were found in the GAS group. Our study indicated a similar result that TCT had a low positivity rate for HPVI ECAs (61.5% for GAS and 55.6% for non-GAS HPVI ECA). Thus, some of these cases may be missed during conventional screening because of negative results from both hrHPV and cytology. As a result, the patient with HPVI ECA is frequently diagnosed at a relatively later stage. Although no significant difference was detected, the TCT results showed higher rate of NILM in MDA than the rate of NILM in non-MDA GAS (63.6% vs 20.0%, *p* = 0.06). This reminds us that MDA might be more prone to be missed and misdiagnosed clinically.

CA19–9 is of great clinical importance in the diagnosis, treatment and prognosis of gastrointestinal malignancies, and it is closely related to disease progression [22–24]. However, elevated serum CA19–9 in ECAs has rarely been reported. Until now, only Nakamural et al. [21] reported that serum CA19–9 in GAS was higher than that in non-GAS HPVI ECAs. They compared the rate of cases with elevated CA19–9 levels, and found it was higher than the rate of levels among non-GAS HPVI ECAs. However, in their report, some cases with hrHPV infection were also included in the non-GAS HPVI ECAs. In our study, we found that the serum CA19–9 level of patients with MDA GAS was significantly higher than that of patients with non-GAS HPVI ECA (161.0 U/ml vs 10.1 U/ml, *p* = 0.001). Although no significant difference was found between MDA and non-MDA GAS (161.0 U/ml vs 22.8 U/ml, *p* = 0.121), we believed that there may be certain testing deviations for this is a retrospective study. While the number of cases with elevated serum CA19–9 was more in MDA than in both non-MDA GAS and non-GAS HPVI ECA (*p* = 0.022 and *p* = 0.006 respectively). It demonstrated that the elevated serum CA19–9 mainly occurred in MDA cases. Thus, it might be an effective tumor marker for the differential diagnosis of MDA, and non-MDA GAS or non-GAS HPVI ECA. As mentioned before, MDA is more prone to be missed by cytology. Taken together, CA19–9 might be an effective tumor marker for clinical diagnosis of GAS, especially for MDA.

Kojima et al. [17] demonstrated higher frequencies of destructive invasive patterns, LVSI, and advanced stage in HPVI ECAs. We further analyzed the stage and pathological features of GAS and non-GAS HPVI ECAs. In our study, GAS cases were more likely to be in the stage above IIB than those of non-GAS HPVI ECAs (54.0% vs 15.4%, *p* = 0.036). Karamurzin et al. [15] reported that 59% of GAS were staged over II, which was a significant difference when compared with HPVA ECA cases. A previous report showed that GAS had always been diagnosed at a more advanced stage than usual type HPVA ECAs [11]. According to our results, GAS cases were more likely to infiltrate into the parametrial and pelvic organs than non-GAS HPVI ECA cases. In addition, there were significant differences in presence of deep stromal invasion and LVSI in GAS compared with non-GAS HPVI ECAs (*p* = 0.002 and *p* = 0.044, respectively). Although no significant difference was found in lymph nodes metastasis between GAS and non-GAS HPVI ECAs, the incidence seems to be higher in GAS (29.4% vs 7.7%).

It has been reported that HPVI ECAs portend worse prognosis than HPVA ECAs, including OS, DFS, and PFS. Kojima et al. [11] demonstrated that patients with HPVI ECA (including 12 GAS and 4 MDA) had significantly decreased 5-year DFS compared with usual type HPVA ECA. In addition, GAS was associated with an increased risk of recurrence compared with non-GAS HPVI ECA. Karamurzin et al. [15] reported that disease specific survival (DSS) at 5 years was 42% for GAS compared with 91% for usual type HPVA ECAs. Few studies have focused on outcomes of GAS and non-GAS HPVI ECAs. We found that the prognosis of GAS was worse than that of non-GAS HPVI ECAs. Compared with GAS patients, patients with non-GAS had an 84% reduction in the risk of relapse (*p* = 0.02), and 90% reduction in the risk of mortality (*p* = 0.03). In our study, the most common postoperative adjuvant therapy was RT combined with CT for GAS, regardless of eligibility according to the SEDLIS criteria by NCCN. The value of adjuvant therapy after surgery needs further investigation.

There has been considerable debate about clinical outcomes of MDA since compared with HPVA ECA. Several studies had indicated its relatively aggressive nature [7]. Nishio et al. [25] reported that more than half the patients died of disease, and only three patients were alive without recurrence after 2 years of follow-up. Karamurzin et al. [15] reported forty cases of GAS including 13 of MDA and 27 of non-MDA GAS subtype and found no clinical and survival difference between MDA and non-MDA GAS. Similar to the results of Karamurzin et al., we found no differences between these two groups, including clinical complaints, tumor size, stage, lymph node metastasis, LVSI, deep stromal invasion, and survival outcomes. This reminded us although MDA was a kind of well differentiated adenocarcinoma, the prognosis was almost as poor as non-MDA GAS.

## Conclusions

Screening for successful diagnosis is difficult for patients with GAS. GAS had different clinical presentation with genital watery discharge compared with non-GAS HPVI ECA cases. Comparison with those of non-GAS HPVI ECAs, GAS cases were more likely to have high risk pathological factors such as deep stromal invasion, LVSI, and advanced stage, with poorer PFS and OS. Serum CA19–9 may be helpful for diagnosis and screening in patients with GAS, especially those with MDA.

## Supplementary Information

**Additional file 1: Supplementary Table 1.** Results of HR-HPV and P16 IHC staining. **Supplementary Table 2.** Treatment and outcomes of HPVI ECAs.

## Data Availability

The data in the current study are not publicly available on account that data came from the medical records where sensitive information is collected, but anonymized information is available from the corresponding author on reasonable request.

## Abbreviations

CCC: clear cell adenocarcinoma
CT: chemotherapy
ECAs: Endocervical adenocarcinomas
FIGO: International Federation of Gynecology and Obstetrics
GAS: gastric adenocarcinoma
HPV: human papillomavirus
HPVA: HPV-associated
HPVI: HPV-independent
H&E: Hematoxylin and eosin
hrHPV: high-risk HPV
IHC: immunohistochemical
LVSI: lymphovascular space invasion
MC: mesonephric carcinoma
MDA: minimal deviation adenocarcinoma
NILM: negative for intraepithelial lesions or malignancy
NOS: not otherwise specified
OS: overall survival
PFS: progression free survival
RT: radiotherapy
TCT: Thinprep cytologic test
WHO: World Health Organization
