# Supplementary material for: Clinicopathological features and outcomes in gastric-type of HPV-independent endocervical adenocarcinomas
Source: BMC Cancer. 2021 Oct 11;21:1095. doi: 10.1186/s12885-021-08792-7 (PMC8507215; doi:10.1186/s12885-021-08792-7)
Supplement: Supplementary file 1 — Additional file 1: Supplementary Table 1. Results of HR-HPV and P16 IHC staining. Supplementary Table 2. Treatment and outcomes of HPVI ECAs. [file 12885_2021_8792_MOESM1_ESM.doc]

**Supplementary Table 1. Results of HR-HPV and P16 IHC staining**

|  | **GAS (n)** | **Non-GAS(n)** | **Total** |
| --- | --- | --- | --- |
| **HR-HPV** |  |  |  |
| NA | 7 | 7 | 14 |
| Negative | 31 | 10 | 41 |
| Positive | 0 | 0 | 0 |
| **P16 IHC staining** |  | |  |
| Negative | 27 | 6 | 33 |
| Patchy Positive | 11 | 11 | 22 |
| Strong Positive | 0 | 0 | 0 |

Supplementary Table 2. Treatments and outcomes of non-HPV adenocarcinoma

|  | **GAS** | **Non-GAS** |
| --- | --- | --- |
| **Treatment (n)** |  |  |
| Surgery | 5 | 2 |
| Radio+Chemo | 2 | 2 |
| Sur.+Ajuvant Therapy | 31 | 13 |
| **Outcomes** |  |  |
| Recurrence case (n) | 16 | 2 |
| Death case (n) | 12 | 1 |
